# Enhanced Catalytic Performance of Sn Single-Atom Doped CuO with Oxygen Vacancies for Efficient Epoxidation of α-Olefins

**DOI:** 10.3390/molecules30051042

**Published:** 2025-02-25

**Authors:** Min Zhang, Gaolei Qin, Pengfei Li, Xiangjie Zhang, Hongying Chang, Ziyu Zhou, Wei Zhao, Xumeng Huang, Kui Tang, Yonghe Ning, Chang Song, Peng He

**Affiliations:** 1 State Key Laboratory of Coal Conversion, Institute of Coal Chemistry, Chinese Academy of Sciences, Taiyuan 030001, China; zhangmin@sxicc.ac.cn (M.Z.); qingaolei21@mails.ucas.ac.cn (G.Q.); zhangxiangjie18@mails.ucas.ac.cn (X.Z.); zhouziyu@sxicc.ac.cn (Z.Z.); zhaowei22@mails.ucas.ac.cn (W.Z.); huangxumeng24@mails.ucas.ac.cn (X.H.); tangkui24@mails.ucas.ac.cn (K.T.); ningyongh@163.com (Y.N.); 2 School of Chemical Engineering, University of Chinese Academy of Sciences, Beijing 100049, China; lipengfei21@mails.ucas.ac.cn (P.L.); changhongying22@mails.ucas.ac.cn (H.C.)

**Keywords:** long chain α-olefins, Mukaiyama epoxidation, oxygen vacancies, electron-rich Cu, synergistic enhancement

## Abstract

Epoxidation of long-chain α-olefins (LAOs) is a process of paramount importance, particularly in the preparation of epoxides. Traditional epoxidation methods, such as the chlorohydrin method and peracid method, suffer from issues such as poor selectivity, by-product formation, and environmental pollution. Mukaiyama epoxidation, with its mild reaction conditions and exceptional selectivity, has attracted widespread attention and considerable research. Transition metal oxide catalysts show potential in the reaction; however, the catalytic efficiency still require substantial improvement due to dilemma of substance activation. In this study, a synergistic enhancement method was employed, achieved through the creation of oxygen vacancies and the electron-rich nature of Cu. The substitution of Cu with Sn in CuO facilitates the creation of oxygen vacancy (Vo), thereby enhancing absorption and activation of O_2_. The conversion for O_2_ activation paves the way for the formation of benzoyl peroxy radicals. Moreover, the interaction between Sn and Cu promotes charge transfer from Sn to Cu, resulting in an electron-rich Cu surface that significantly accelerates the dehydrogenation of benzaldehyde. The synergistic enhancement protocol exhibits near-quantitative performance, delivering an oxide yield of 92.9%. This study introduces an innovative dual-promotion catalytic strategy for Mukaiyama epoxidation utilizing readily available O_2_, providing profound insights into the optimization design of transition metal oxide catalysts and beyond.

## 1. Introduction

Epoxidation of olefins is a key reaction widely used in the synthesis of epoxides and functionalized molecules, with significant importance in the industrial production of polymers, surfactants, and the enhancement of lubricating oil performance [1,2]. Long-chain α-olefin (LAO) epoxides, owing to their unique chemical structures and reactivity, are critical materials in these applications [3,4]. Compared to cyclic and aromatic olefins, the electron-deficient characteristics of LAOs markedly diminish their reactivity, reducing their susceptibility to activation [5,6]. This inherent structural limitation restricts their interaction with electrophilic reagents, thereby hindering the efficient conversion of LAOs into epoxide [7,8]. Therefore, identifying the optimal reaction pathway with effective and mild conditions has been a critical objective for epoxidation of LAOs [9,10].

Traditional olefin epoxidation methods, such as the chlorohydrin process and peracid epoxidation, have shown high conversion rates in some cases but are often associated with environmental pollution, by-product generation, and equipment corrosion [11]. The chlorohydrin process involves the reaction of olefins with chlorine and water to generate chlorohydrin intermediates, which are then further oxidized or treated with base to yield epoxides [12,13]. However, this method suffers from poor selectivity and environmental concerns. Although peracid epoxidation efficiently converts olefins to epoxides, issues such as by-product formation, high costs of peracids, and environmental safety concerns limit its widespread application [14,15].

**Scheme 1:**
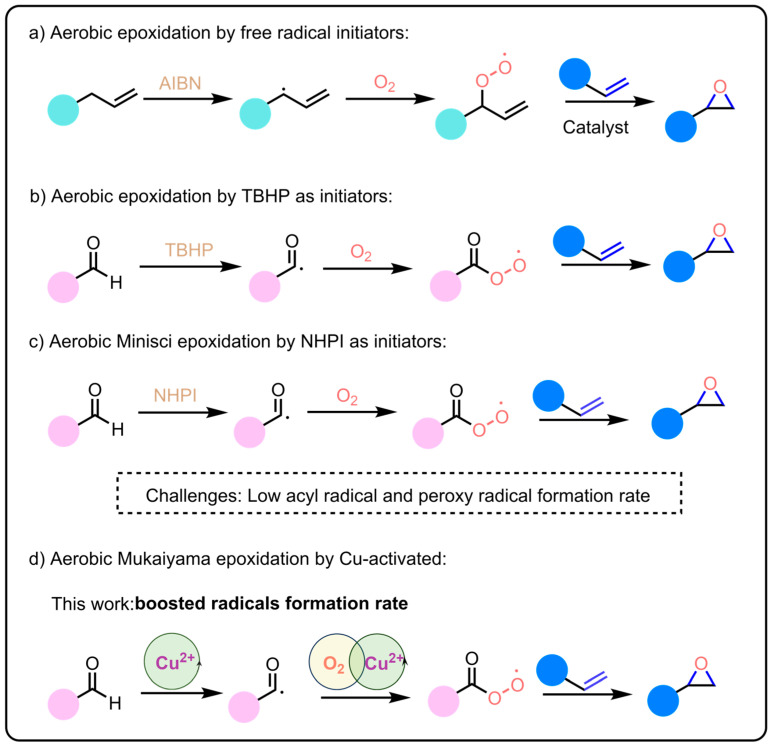
Epoxidation of olefins with molecular oxygen.

In contrast to traditional epoxidation processes, leveraging oxygen as the oxidant presents multiple advantages [16,17]. Primarily, oxygen—being the most abundant and cost-effective oxidant—can substantially lower production costs [18,19]. However, free radical-initiated epoxidation methods using oxygen, while offering certain reaction efficiency, still face limitations in practical applications due to difficulties in controlling the reaction, poor selectivity, and by-product formation [20,21,22,23,24] (Scheme 1). Recently, transition metal-catalyzed approaches, particularly those utilizing homogeneous metal complexes with oxygen as the oxidant, have garnered significant attention for their advantages in selectivity, mild reaction conditions, and environmental sustainability [25]. Nonetheless, these methods are not without their challenges, including catalyst recovery and deactivation—issues commonly encountered in homogeneous catalysis [26,27].

The Mukaiyama epoxidation, as an emerging heterogenous method, addresses the aforementioned challenges by utilizing transition metal oxide catalyze an aldehyde and oxygen to generate acylperoxy radicals for olefin epoxidation. This reaction is suitable for a wide range of olefins, including LAOs, aromatic olefins, and complex asymmetric olefins, and it exhibits selectivity, particularly for LAOs. Despite the high selectivity of the Mukaiyama epoxidation in many olefin reactions, the catalytic activity remains a bottleneck. In particular, for complex substrates or low-concentration reactant systems, the catalyst may require longer reaction times or higher aldehyde usage to achieve high conversion rates. This may be ascribed to the relatively low generation rates of acyl radicals and acylperoxy radicals [28].

To enhance catalyst activity, various optimization strategies have been proposed, especially in the design of transition metal catalysts [29,30]. This paper presents an innovative strategy based on the synergistic enhancement effect in CuO catalysts to efficiently catalyze olefin Mukaiyama epoxidation. Studies have shown that metal oxide catalysts often suffer from insufficient active sites and slow reaction kinetics when converting aldehydes to acylperoxy radicals, leading to low overall reaction efficiency. Oxygen vacancy (Vo), a critical defect in determining the catalytic performance of transition metal oxides, has been demonstrated to significantly enhance the Lewis acidity and catalytic activity of the catalyst. By introducing oxygen vacancies, the adsorption of reactants and intermediates on the catalyst surface is enhanced, accelerating the reaction process.

In this work, we employed Sn single-atom doped CuO catalysts to significantly enhance the charge density and oxygen vacancy formation in CuO. The doping of Sn enhanced the charge density of the CuO catalyst, improving its ability to capture the aldehyde C-H bond to form acyl radicals, while also significantly increasing the number of oxygen vacancies, which promoted the adsorption and activation of oxygen on the catalyst surface, facilitating the reaction between O_2_ and acyl radicals to generate acylperoxy intermediates and ultimately accelerating the epoxidation of olefins. Experimental results demonstrated that the Vo synergized electron-rich Cu in CuO catalyst exhibited outstanding catalytic performance in the Mukaiyama epoxidation reaction, achieving near-perfect product yield (92.9%). Experiments and theoretical calculation results further confirmed that this excellent catalytic activity arose from significant changes in the electronic state of the active sites and oxygen vacancy.

## 2. Results and Discussion

### 2.1. Characterization of Catalysts

**Figure 1:**
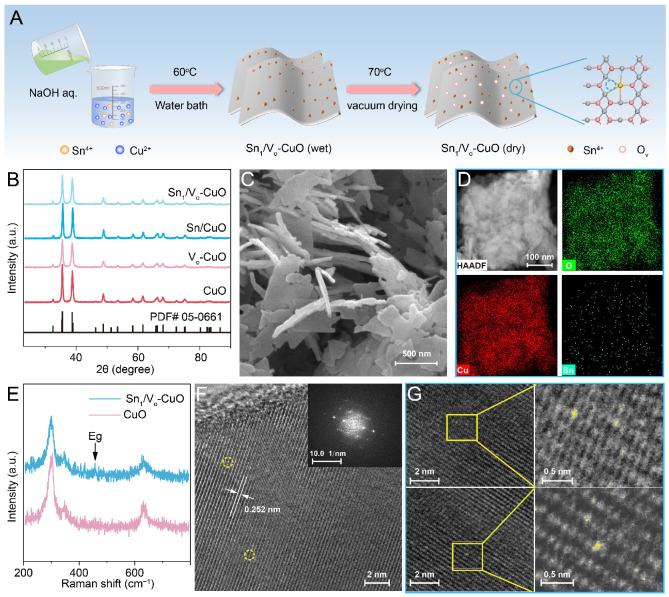
(**A**) Schematic illustration of the synthesis process for Sn_1_/V_o_-CuO. (**B**) XRD patterns of the as-synthesized Four catalysts. (**C**) SEM images of Sn_1_/V_o_-CuO nanosheets. (**D**) The EDS mapping of as-prepared Sn_1_/V_o_-CuO nanosheets. (**E**) Raman spectra of Sn_1_/V_o_-CuO and CuO samples. (**F**) High-Angle Annular Dark-Field Scanning Transmission Electron Microscopy (HAADF-STEM) images of Sn_1_/V_o_-CuO. The inset in (**F**) is its fast Fourier transform (FFT) pattern. In image (**F**), some individual Sn atoms are marked with yellow dashed circles. (**G**) A STEM diagram of Sn_1_/V_o_-CuO nanosheet; the images in the yellow rectangular area are the transformed STEM images of Sn_1_/V_o_-CuO nanosheets.

As shown in Figure 1A, Sn single-atom-doped CuO nanosheets with abundant oxygen vacancies were synthesized via a simple one-pot method. The process is as follows: Cu(CH_3_COO)_2_·H_2_O was first dissolved in deionized water, and then SnCl_4_·5H_2_O solution was gradually added. Next, under magnetic stirring, a 100 mL NaOH solution was slowly added to the mixed precursor solution. Finally, the mixture was kept at 60 °C for 3 h to obtain Sn_1_/V_o_-CuO nanosheets, which were recovered by centrifugation and drying under vacuum. The synthesis of V_o_-CuO nanosheets followed the same procedure, but without the addition of Sn precursor. The crystal structure of the synthesized products was characterized by powder X-ray diffraction (XRD). The XRD patterns of the sample powders are shown in Figure 1B. The diffraction peaks of all four catalysts are nearly identical, and all the peaks correspond well with the standard CuO spectrum (PDF# 05-0661). These peaks are attributed to CuO with a pure monoclinic symmetry and space group C2/c.

The scanning electron microscopy (SEM) characterization results of the samples are shown in the Figure 1C and Figure S1. All the samples exhibit sheet-like structures, with no significant morphological differences among the four samples. This indicates that the doping of Sn and the calcination process have minimal impact on the morphology of the CuO sheets. Elemental mapping (Figure 1D and Figure S2) and energy dispersive spectroscopy (EDS) analyses (Figure S3) show the uniform distribution of Cu and Sn in the sample, indicating that Sn atoms are evenly distributed throughout the structure. The Raman spectra of CuO and Sn_1_/V_o_-CuO samples are shown in Figure 1E. Three distinct peaks at 297, 328, and 630 cm^−1^ are observed, corresponding to the Ag, Bg1, and Bg2 modes of CuO nanosheets, respectively [31,32]. For the Sn_1_/V_o_-CuO sample, these peaks shift to higher wavenumbers, and a new weak peak appears at 457 cm^−1^, attributed to the Eg mode of SnO_2_ [33]. The results imply the existence of Sn in the catalyst. Further structural analysis of the Sn_1_/V_o_-CuO nanosheets was conducted using high-angle annular dark-field scanning transmission electron microscopy (HAADF-STEM). Figure 1F displays clear lattice fringes with a spacing of 0.252 nm, corresponding to the (−111) plane of CuO, which is consistent with the results from the Fourier transform analysis (the inset in Figure 1F). In Figure 1G, these spots can be plausibly ascribed to single Sn atoms. This is because the atomic number of Sn is substantially larger than that of Cu or O, which, consequently, leads to a more prominent and brighter manifestation in high-angle annular dark-field scanning transmission electron microscopy (HAADF-STEM). The magnified HAADF-STEM images offer additional and more conclusive evidence, firmly corroborating the successful formation of discretely isolated Sn atoms.

X-ray photoelectron spectroscopy (XPS) analysis was performed to investigate the composition and valence states of the Sn_1_/V_o_-CuO catalyst. The full XPS spectrum of the Sn_1_/V_o_-CuO sample shows peaks corresponding to Cu, O, and Sn elements (Figure S4A), consistent with the results from elemental mapping analysis. In the high-resolution Cu 2p spectrum (Figure S4B), two peaks at 934.0 eV and 953.9 eV correspond to the spin-orbit splitting features of Cu 2p_3/2_ and Cu 2p_1/2_ in CuO, indicating that Cu^2+^ is the predominant oxidation state in this material. Additionally, a weak peak at 933.0 eV was also observed in the XPS spectrum. This peak is assigned to Cu^+^ in the catalyst, suggesting the presence of oxygen vacancies in Sn_1_/V_o_-CuO. Perhaps, Cu^+^ or Cu^0^ species exist in Sn_1_/V_o_-CuO. However, it is hard to distinguish them from the Cu 2p spectrum due to the similarity in the binding energies. Therefore, the Cu Auger LMM spectrum (Figure S4C) was also measured to gain more insights into the electronic structures and surface state, which indicates the coexistence of Cu^+^ and Cu^0^ species on Sn_1_/V_o_-CuO. In the Sn 3d spectrum (Figure S4D), characteristic Sn 3d_3/2_ and Sn 3d_5/2_ peaks are observed at binding energies of 486.6 eV and 495.0 eV, respectively, confirming that Sn is in the +4 oxidation state. The atomic ratio of Cu to Sn within the as-prepared Sn_1_/V_o_-CuO nanosheet was precisely determined to be 96.85:3.15 through quantitative analysis, which was based on the experimental data acquired from inductively coupled plasma optical emission spectroscopy (ICP-OES), as presented in Table S1.

**Figure 2:**
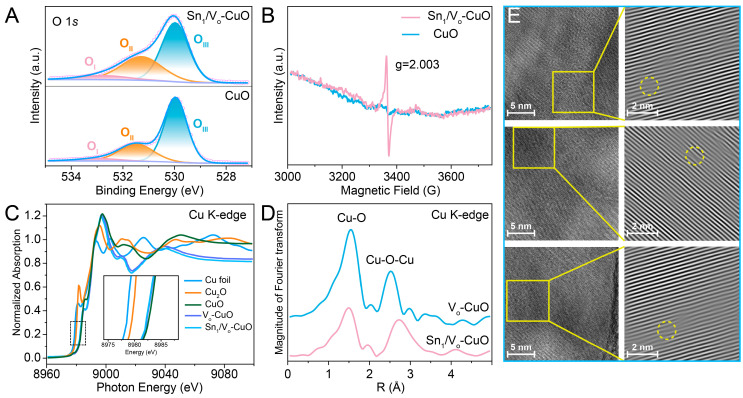
(**A**) High-resolution XPS spectra of O 1s. (**B**) EPR spectra of Sn_1_/V_o_-CuO and CuO samples. (**C**) Cu K-edge XANES spectra of Vo-CuO and Sn_1_/V_o_-CuO as well as Cu foil, Cu_2_O, and commercial CuO. (**D**) Fourier-transformed EXAFS spectra of V_o_-CuO and Sn_1_/V_o_-CuO at the Cu K-edge. (**E**) HRTEM images and the corresponding inverse fast Fourier transform (IFFT) image of Sn_1_/V_o_-CuO catalysts. In image (**E**), some oxygen vacancy are marked with yellow dashed circles.

O 1s XPS analysis was performed on the synthesized material. As shown in Figure 2A, a characteristic peak at 530.0 eV is attributed to lattice oxygen (Cu−O−Cu, O_III_). The peaks at 531.4 eV are attributed to surface-adsorbed oxygen (O_2_^2−^ or O_2_^−^), which is associated with the presence of oxygen vacancies (O_II_). Additionally, peaks are observed at 532.7 eV, corresponding to adsorbed H_2_O and hydroxyl groups (O_I_) on the catalyst surface. To further verify the presence of oxygen vacancies in the Sn_1_/V_o_-CuO catalyst, electron paramagnetic resonance (EPR) measurements were conducted. As shown in Figure 2B, the Sn_1_/V_o_-CuO sample exhibits a distinct EPR signal near g = 2.003, in contrast to the flat curve collected from CuO [34]. This signal is attributed to electrons trapped at oxygen vacancies. The EPR experiment strongly supports the existence of abundant oxygen vacancies in the Sn_1_/V_o_-CuO structure. The Cu K-edge XANES spectra of the obtained V_o_-CuO and Sn_1_/V_o_-CuO, along with those of commercial CuO, Cu_2_O, and Cu foil, are shown in Figure 2C. The Cu in Sn_1_/V_o_-CuO has a lower edge position and lower line intensity, indicating that it is partly reduced due to the incorporation of Sn. Fourier-transformed EXAFS (Figure 2D) shows that the coordination number of oxygen for Cu in Sn_1_/V_o_-CuO is smaller compared to that in V_o_-CuO (Table S6), demonstrating the formation of more oxygen vacancies in the composite system [35]. High-Resolution Transmission Electron Microscopy (HRTEM) images, along with their corresponding Inverse Fast Fourier Transform (IFFT) images, were presented in order to provide an intuitive visualization of the existence of V_o_, as depicted in Figure 2E [36].

### 2.2. Catalytic Performance

In this study, the performance of Sn_1_/V_o_-CuO catalyst in 1-octene Mukaiyama epoxidation was evaluated. Reaction conditions were optimized by investigating the impacts of temperature, the molar ratio of benzaldehyde to 1-octene, and solvent type. Aldehyde was crucial for acyl peroxy radical formation, without which the reaction hardly proceeded. Figure S5 shows the volcano-type relationship between the molar ratio and catalytic activity/selectivity. Initially, increasing aldehyde concentration significantly enhances the molecular collision frequency between alkene and aldehyde reactants, thereby improving both catalytic activity and selectivity. This positive correlation continues until an optimal concentration is reached. Beyond this threshold, however, the elevated aldehyde concentration preferentially facilitates thermodynamically favorable aldehyde dimerization side reactions, which compete with the main catalytic cycle. This dual concentration effect, consequently, results in a characteristic volcano-shaped profile when plotting the aldehyde-to-catalyst molar ratio against catalytic performance metrics, including both conversion efficiency and product selectivity. The optimal ratio of benzaldehyde to 1-octene was 5:1. Temperature studies (30–90 °C) indicated that the best yield was at 60 °C, with conversion and yield peaking then decreasing due to side reactions (Figure S6). Acetonitrile was the preferred solvent, while methanol and DMSO hampered the reaction (Figure S7). In summary, the optimal conditions for Sn_1_/V_o_-CuO were a 5:1 molar ratio, 60 °C, and acetonitrile. Under optimized conditions, the impact of the reaction time on the catalyst’s performance was assessed.

**Figure 3:**
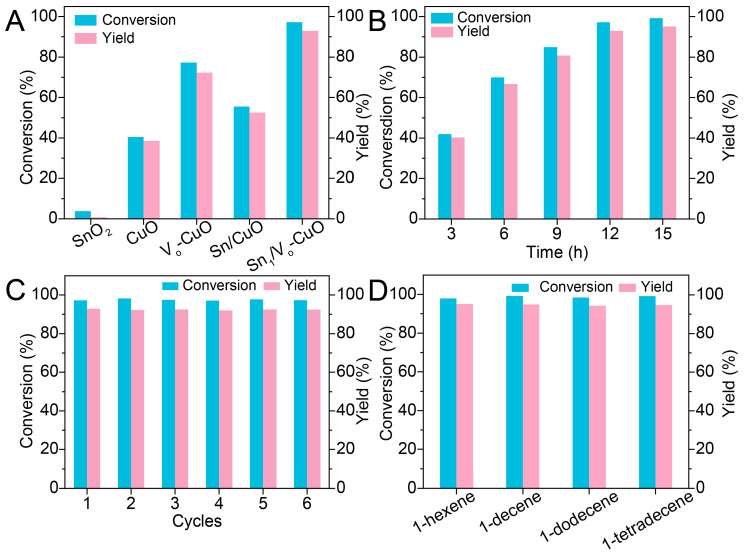
(**A**) Conversion and yield towards epoxide of SnO_2_, CuO, V_o_-CuO, Sn/CuO, Sn_1_/V_o_-CuO catalysts. Reaction condition: 10 mg catalyst, 1 mmol 1-octene, 5 mmol benzaldehyde, 5 mL acetonitrile, 60 °C, 12 h, O_2_ balloon. (**B**) Effect of reaction time for epoxidation of 1-octene with Sn_1_/V_o_-CuO. Reaction condition: 10 mg catalyst, 1 mmol 1-octene, 5 mmol benzaldehyde, 5 mL acetonitrile, 60 °C, O_2_ balloon. (**C**) Recycling of Sn_1_/V_o_-CuO for 1-octene epoxidation. Reaction condition: 10 mg catalyst, 1 mmol 1-octene, 5 mmol benzaldehyde, 5 mL acetonitrile, 60 °C, 12 h, O_2_ balloon. (**D**) The results for the epoxidation of various olefins. Reaction conditions: 10 mg catalyst, 1 mmol olefin, 5 mmol benzaldehyde, 5 mL acetonitrile, 60 °C, 12 h, O_2_ balloon (all specific values are in Tables S1–S4).

Under the specified conditions, a comprehensive evaluation of the performance of various catalysts was carried out. As depicted in Figure 3A, when SnO_2_ was employed as the catalyst, virtually no epoxide was generated. In contrast, CuO achieved a yield of 33.2% for 1,2-epoxy-octane. Notably, the V_o_-CuO catalyst demonstrated a relatively higher yield of 73.1% for 1,2-epoxy-octane. However, the Sn_1_/V_o_-CuO catalyst exhibited remarkably superior catalytic performance, attaining an impressive 92.9% yield of 1,2-epoxy-octane and a 97.2% conversion rate of 1-octene, outperforming both CuO and Sn/CuO catalysts. This enhanced performance of Sn_1_/V_o_-CuO can likely be attributed to its abundant oxygen vacancies. These findings clearly indicate that the strategy of simple atomic doping to augment the number of oxygen vacancies can significantly enhance the performance of the Mukaiyama epoxidation reaction, leading to more than a twofold increase in the yield of 1,2-epoxy-octane. It is noteworthy that the Sn_1_/V_o_-CuO catalyst demonstrates relatively low turnover frequency (TOF) and turnover number (TON) values, specifically 4 h^−1^ and 48, respectively. This suboptimal catalytic performance can be primarily ascribed to two factors: (1) the existence of a substantial proportion of bulk Cu sites that remain inactive during the catalytic process and (2) the presence of inefficient surface Cu sites. Consequently, enhancing the utilization efficiency of Cu species emerges as a crucial direction for future research endeavors. Figure 3B shows the changes in 1-octene conversion and 1,2-epoxy-octane yield over time. The yield of 1,2-epoxy-octane of Sn_1_/V_o_-CuO rose continuously in the first 15 h, with the rate slowing after 12 h. Figure S8 illustrates the kinetic profile of the catalytic reaction. The kinetic analysis reveals the absence of a significant induction period, which is in good agreement with our proposed reaction mechanism. This observation supports the hypothesis that Cu(II) species functions as the catalytically active site without requiring additional activation processes. Moreover, the catalytic system maintains consistent selectivity throughout the reaction duration, as evidenced by the time-dependent product distribution analysis.

The reusability and stability of a catalyst are crucial parameters for its practical application. Under similar reaction conditions, the Sn_1_/V_o_-CuO catalyst was subjected to six consecutive cycles of testing to evaluate its stability. As illustrated in Figure 3C, after six cycles, the catalytic activity and the yield of 1,2-epoxide of the recovered catalyst remained essentially unchanged, signifying the outstanding stability of the Sn_1_/V_o_-CuO catalyst. Furthermore, an analysis of the liquid phase separated from the reaction mixture after the catalytic reaction revealed the absence of copper, indicating that no leaching of copper from the catalyst occurred during the reaction process. In addition, we performed XRD (Figure S9) and SEM (Figure S10) characterizations on the structure and morphology of the catalyst before and after the reaction. It can be observed from the Figures S9 and S10 that both the structure and the morphology of the catalyst remained substantially unaltered before and after the reaction. The Sn_1_/V_o_-CuO catalyst demonstrates excellent substrate adaptability toward long-chain α-olefins, as evidenced by its consistent catalytic performance across a series of linear aliphatic olefins with varying carbon chain lengths (Figure 3D).

### 2.3. Study of the Reaction Mechanism

Our previous investigations have determined that the copper-catalyzed Mukaiyama epoxidation occurs through two essential steps [37]: aldehyde oxidation and alkene epoxidation. Significantly, the aldehyde oxidation step is the rate-controlling step for the overall reaction. In this stage, the aldehyde is oxidized on the catalyst surface, generating an acyl radical, which then reacts with oxygen to form an acyl peroxy radical. This acyl peroxy radical is generally considered the reactive oxygen species that drives the subsequent epoxidation of the alkene [38].

**Figure 4:**
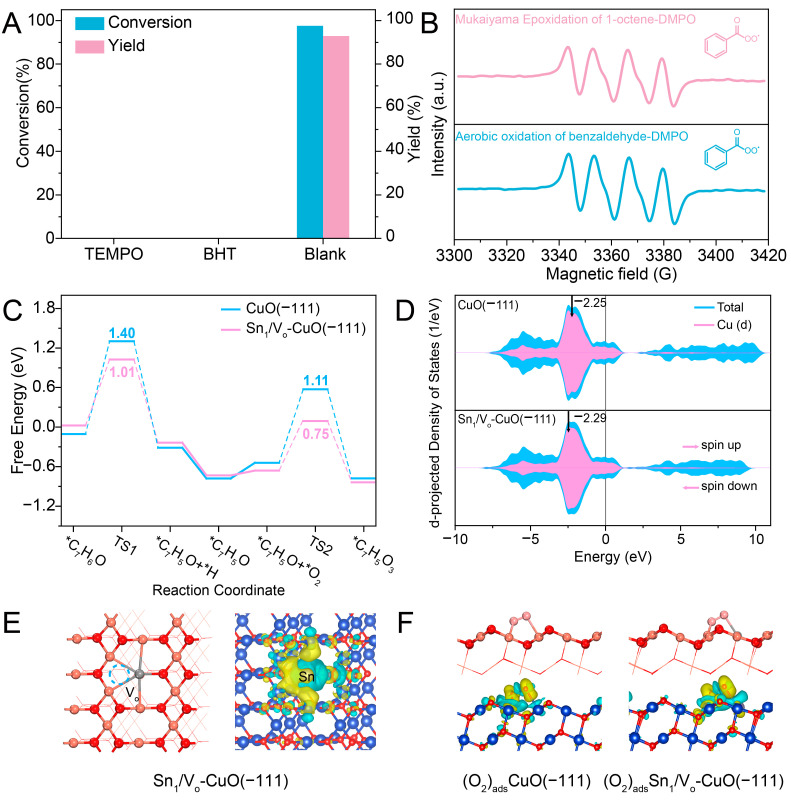
(**A**) Active radicals quenching experiments for epoxidation of 1-octene catalyzed by the Sn_1_/V_o_-CuO. (**B**) EPR spectra of Mukaiyama epoxidation and aerobic oxidation of benzaldehyde. (**C**) Free energy diagram for the formation process of benzoyl peroxy radical on CuO(−111) and Sn_1_/V_o_-CuO(−111), the asterisk (*) represents the states of adsorption on the catalyst surface. (**D**) Projected density of states (pDOS) plots of the d-orbitals for the Cu atoms of CuO(−111) and Sn_1_/V_o_-CuO(−111). The vertical dashed and solid lines represent the location of d-band center and Fermi level, respectively. (**E**) Top view of charge density difference for Sn_1_/V_o_-CuO(−111) after Sn doping (O: red, Cu: orange and blue, Sn: grey). (**F**) Side view of charge density difference for O_2_ adsorption on CuO(−111) and Sn_1_/V_o_-CuO(−111) (O: red, Cu: orange and blue, Sn: grey).

Building upon our prior knowledge of the reaction steps, we initiated a comprehensive exploration into the epoxidation reaction mechanism. At the outset, free radical quenching experiments were carried out by incorporating two equivalents of either TEMPO or BHT, which are well-known radical scavengers, into the reaction system. In both experimental scenarios, the complete absence of epoxide formation was noted (Figure 4A). This observation provided conclusive evidence, unequivocally confirming that the reaction indeed progresses through a free radical pathway. It is noteworthy that TEMPO has the ability to scavenge all types of radicals, while BHT is mainly effective in quenching carbon-centered radicals. The acyl radical is a type of carbon-centered radical. The lack of reaction upon the addition of BHT might be due to the fact that BHT scavenged the acyl radical, preventing the reaction from proceeding. This quenching experiment also indirectly verifies the existence of the acyl radical [39].

In the Sn_1_/V_o_-CuO-catalyzed 1-octene Mukaiyama epoxidation, electron paramagnetic resonance (EPR) spectroscopy was utilized to monitor the acylperoxy radical, which is the active species involved in the epoxidation process on the Sn_1_/V_o_-CuO catalyst (Figure 4B). The presence of the acyl peroxy radical was successfully detected in both the Mukaiyama epoxidation system and the aerobic oxidation system of aldehydes. Furthermore, we also detected the radicals in the reaction system under strictly anaerobic conditions (Figure S11). The spectrum obtained from our electron paramagnetic resonance (EPR) test was similar to that detected in the anaerobic oxidation system of aldehydes, exhibiting the characteristic peaks of carbon-centered radicals. This result indicates that acyl radicals are likely to be generated within the reaction system [24].

To gain a deeper understanding of the reaction mechanism and the role of the catalyst components, we undertook computational studies. In the 1-octene Mukaiyama epoxidation reaction, the use of benzaldehyde as a sacrificial agent to activate O_2_ and generate the reactive benzoyl peroxy radical is of crucial importance. Given that the formation of the benzoyl peroxy radical is kinetically slow and has a significant impact on the overall epoxidation rate, we incorporated Density Functional Theory (DFT) calculations of this process into our research. The corresponding configurations and free energy data of the reaction process are presented in the Supporting Information (Figures S12–S15).

The free energy diagrams for the dehydrogenation of benzaldehyde and the interaction of the benzoyl radical with O_2_ on CuO(−111) and Sn_1_/V_o_-CuO(−111) are shown in Figure 4C. It is clear that the adsorption of benzaldehyde on the CuO(−111) surface is weaker than its adsorption on the Sn_1_/V_o_-CuO(−111) surface. This can be mainly attributed to the fact that Sn doping causes a slight shift in the band center of Cu away from the Fermi energy level (−2.29 eV vs. −2.25 eV, Figure 4D). However, the energy barrier for benzaldehyde dehydrogenation on the Sn_1_/V_o_-CuO(−111) surface is much lower than that on the CuO(−111) surface. As illustrated in Figure 4E, Sn doping leads to the transfer of electrons from Sn to CuO, resulting in a significantly increased electron density of nearby Cu atoms. This, in turn, promotes the dehydrogenation of benzaldehyde on the Sn_1_/V_o_-CuO(−111) surface.

Furthermore, in addition to enhancing benzaldehyde dehydrogenation, Sn doping also facilitates the formation of O vacancies on the CuO(−111) surface. On the unmodified CuO(−111) surface, the oxygen vacancy formation energy Ef(V_o_) is as high as 1.99 eV, making it difficult to form. However, after Sn doping, this energy is reduced to 1.29 eV (Figure S13). As shown in Figure 4F, there is a significant charge transfer from the Sn_1_/V_o_-CuO(−111) surface to adsorbed O_2_. The greater amount of charge transfer indicates a stronger adsorption strength of O_2_ on Sn_1_/V_o_-CuO(−111). This enhanced activation of O_2_ on the Sn_1_/V_o_-CuO(−111) surface reduces the energy barrier for the interaction between O_2_ and the benzoyl radical from 1.11 eV on the CuO(−111) surface to 0.75 eV on Sn_1_/V_o_-CuO(−111).

**Scheme 2:**
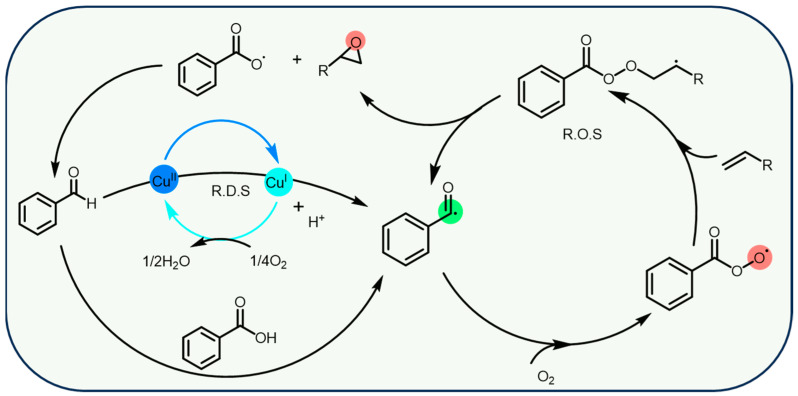
Proposed radical reaction mechanism for olefin epoxidation over Sn_1_/V_o_-CuO catalysts.

In summary, Sn doping plays a multifaceted and important role in the Mukaiyama epoxidation reaction. It not only alters the surface charge distribution of CuO, thereby facilitating the dehydrogenation of benzaldehyde, but also enables the formation of O vacancies, which in turn activates O_2_ and accelerates the generation of benzoyl peroxy radicals. These combined effects result in the superior catalytic performance of the Sn_1_/V_o_-CuO catalyst compared to the undoped CuO, as evidenced by the increased yield and conversion rates observed in the 1-octene epoxidation reaction. This comprehensive understanding of the reaction mechanism and the role of Sn doping provides a solid basis for further optimizing and developing more efficient catalytic systems for the Mukaiyama epoxidation reaction. Based on the above experimental results, theoretical calculations, and relevant literature [40,41], a reasonable reaction mechanism (Scheme 2) is proposed. Firstly, under the catalysis of divalent copper, benzaldehyde undergoes dehydrogenation to generate a benzoyl radical, which is the rate-determining step (R.D.S) of the entire reaction. Simultaneously, divalent copper is reduced to monovalent copper. Subsequently, monovalent copper is oxidized back to divalent copper by oxygen, thus completing the catalyst cycle. The generated benzoyl radical combines with the oxygen adsorbed on the oxygen vacancies to form a benzoyl peroxy radical. Subsequently, the benzoyl peroxy radical, as a reactive oxygen species (R.O.S), oxidizes the alkene to form an epoxide, while benzaldehyde is oxidized to benzoic acid.

## 3. Materials and Methods

### 3.1. Material Synthesis

All chemicals used in this study were analytical grade reagents provided by Aladdin Chemical Co. (Shanghai, China). The reagents included 1-Octene (C_8_H_14_, 99%), benzaldehyde (C_7_H_6_O, ≥99%), acetonitrile (CH_3_CN, AR), *n*-nonane (C_9_H_20_, ≥99%), copper (II) acetate monohydrate (Cu(CH_3_COO)_2_·H_2_O, 99.0%), tin (IV) chloride pentahydrate (SnCl_4_·5H_2_O, 99.0%), and sodium hydroxide (NaOH).

Synthesis of Sn_1_/V_o_-CuO and CuO: In total, 4 g (10 mmol) of copper acetate monohydrate (Cu(CH_3_COO)_2_·H_2_O, 99.0%) was added to 60 mL of deionized water (DI) and stirred at room temperature until the Cu(CH_3_COO)_2_·H_2_O solution became completely clear. Then, a solution of 200 mg of tin tetrachloride pentahydrate (SnCl_4_·5H_2_O, 99.0%) dissolved in 40 mL of DI water was slowly added to the above solution. Under magnetic stirring, 200 mL of 2 mol/L NaOH aqueous solution was slowly added to the precursor solution under agitation. The mixture was then stirred at 60 °C for 3 h. After the reaction, the black precipitate was separated by centrifugation and collected. The precipitate was washed several times with deionized water and ethanol to remove impurities. Finally, the sample was dried at 70 °C under vacuum overnight to obtain the catalyst, denoted Sn_1_/Vo-CuO. The catalyst prepared in a similar manner but without Sn precursor was denoted Vo-CuO.

Synthesis of Sn/CuO and CuO: The Sn_1_/V_o_-CuO and V_o_-CuO catalysts were placed in a muffle furnace and heated in air at a ramping rate of 5 °C/min to 350 °C, where they were maintained for 5 h. After the heating process, the catalysts were allowed to cool naturally to room temperature, yielding the Sn/CuO and CuO catalysts.

### 3.2. Characterizations

The crystallographic information of the Sn_1_/V_o_-CuO samples was collected by X-ray diffraction (XRD, Bruker D8 Advance, Bruker, Karlsruhe, Germany) using Cu-Kα radiation (40 kV, 40 mA). This analysis provided insight into the phase composition and crystalline structure of the samples.

The morphology and detailed microstructure of the as-prepared samples were examined using field emission scanning electron microscopy (SEM, FEI QUANTA 400, FEI Company, Hillsboro, OR, USA) and high-angle annular dark-field scanning transmission electron microscopy (HAADF-STEM, Talos F200X, FEI Company, USA), both of which were equipped with energy-dispersive X-ray (EDX) detectors.

The surface composition and chemical states of the samples were investigated using X-ray photoelectron spectroscopy (XPS, K-Alpha, Thermo Fisher Scientific, Waltham, MA, USA), which allowed for the determination of the elemental composition and oxidation states of the materials.

Raman spectra were recorded using a Raman spectrometer (Raman, LabRam HR800, HORIBA, Les Ulis, France) with a 532 nm laser excitation wavelength, providing information about the vibrational modes and structural characteristics of the samples.

Inductively coupled plasma optical emission spectroscopy (ICP-OES, Perkin Elmer Optima 2100DV, PerkinElmer, Waltham, MA, USA) was employed to analyze the Sn doping content in the Sn_1_/V_o_-CuO nanosheets, providing quantitative data on the element concentrations in the samples.

Electron paramagnetic resonance (EPR, EMX–Plus, Bruker, Germany) spectroscopy was used to identify and quantify the oxygen vacancies in the as-prepared samples, offering insights into the role of these defects in the catalytic properties. In the EPR spin trap experiments, 5,5-Dimethyl-1-pyrroline N-oxide (DMPO) was utilized as a spin-trapping reagent to investigate the radicals present in the reaction mixture. Typically, 20 μL of DMPO was incorporated into a 1 mL mixture, which consisted of 500 μL of the reaction solution and 500 μL of methanol, to generate a spin-trapping composite. Subsequently, an aliquot of this composite was transferred into a 50 μL tubing capillary tube (intra MARK, Cologne, Germany) and then inserted into a quartz X-band EPR tube. For the purpose of quantitatively comparing the EPR signal intensities, an identical amount of the suspension within the capillary was employed during the testing process.

### 3.3. Catalytic Test

The catalytic aerobic epoxidation of 1-octene was carried out in a 25 mL Schlenk tube. First, the reaction tube containing the catalyst powder was degassed and flushed three times with O_2_ to remove any residual air and fill the tube with oxygen. Then, a solvent mixture consisting of 1-octene (1 mmol), benzaldehyde (5 mmol), and acetonitrile (5 mL) was injected into the reaction tube. The system was bubbled with O_2_ for 30 min prior to the injection of reactants. The reaction mixture was stirred with a magnetic stirrer and placed at 60 °C, with O_2_ balloon for 12 h. After the reaction was complete, the system was allowed to cool to room temperature, and the catalyst was separated by centrifugation. The liquid products were analyzed by pre-calibrated gas chromatography (Agilent 7890B, Santa Clara, CA, USA) using a flame ionization detector (FID) and an HP-5 column, with *n*-nonane as an internal standard. The conversion of feedstocks and product yields were calculated based on the GC analysis using the following equations:

(1) $$Conversion=\frac{\left(mol\;of\;initial\;olefin-mol\;of\;unreacted\;olefin\right)}{mol\;of\;initial\;olefin}\times 100\%$$

(2) $$Selectivity=\frac{mol\;of\;produced\;epoxide}{\left(mol\;of\;initial\;olefin-mol\;of\;unreacted\;olefin\right)}\times 100\%$$

(3) $$Yield=\frac{mol\;of\;produced\;epoxide}{mol\;of\;initial\;olefin}\times 100\%$$

### 3.4. Catalyst Recycling Experiment

The recovered catalyst underwent a washing process with acetonitrile for three times, subsequent to which it was subjected to centrifugal separation. Thereafter, the recovered catalyst was dried at a temperature of 60 °C for a duration of 12 h and was then utilized for the subsequent run.

## 4. Conclusions

In summary, this study has proposed a novel and highly effective approach for the Mukaiyama epoxidation of olefins, leveraging the combined power of oxygen vacancies and electron-rich Cu. Conventional metal-based catalysts typically encounter limitations such as low active site density and sluggish reaction kinetics, leading to suboptimal overall reaction efficiency. In response to these issues, we engineered an optimized catalyst by doping CuO with Sn, which substantially augmented the charge density of Cu nearby Sn and facilitated the formation of oxygen vacancies. This Sn doping strategy not only augmented the electronic density of CuO, thereby facilitating the conversion of benzaldehyde into acyl radicals, but also induced the generation of oxygen vacancies that enhanced the adsorption of oxygen. These combined effects accelerated the formation of acyl peroxy radicals and expedited the epoxidation reaction. Experimental findings conclusively demonstrated that the Sn single-atom doped CuO catalyst exhibited outstanding catalytic performance in the Mukaiyama epoxidation of 1-octene, attaining a remarkable product yield of up to 92.9%. Characterization methodologies and density functional theory (DFT) calculations further corroborated that the optimization of the electronic states at the active sites and the enhanced adsorption of oxygen were pivotal factors in enhancing the catalytic performance. This research not only offers an efficient and environmentally sustainable catalytic system for olefin epoxidation but also imparts novel perspectives on the design and optimization of transition metal oxide catalysts through doping strategies. This refined synergistic enhancement strategy can be applied to regulate metal oxides, offering a new approach in the design of advanced catalysts.

## Data Availability

Data will be made available on request.

## Supplementary Materials

The following supporting information can be downloaded at: https://www.mdpi.com/article/10.3390/molecules30051042/s1, Figure S1: SEM images of (A) CuO, (B) V_o_-CuO, (C) Sn/CuO nanosheets; Figure S2: EDS spectrum of the as-synthesized Sn/CuO; Figure S3: (A) XPS survey spectrum of Sn_1_/V_o_-CuO nanosheets. (B) High-resolution XPS spectrum of Cu 2p collected from Sn_1_/V_o_-CuO. (C) LMM Auger spectra of Cu for Sn_1_/V_o_-CuO. (D) High-resolution XPS spectrum of Sn 3d for Sn_1_/V_o_-CuO; Figure S4: (A) XPS survey spectrum of Sn_1_/V_o_-CuO nanosheets. (B) High-resolution XPS spectrum of Cu 2p collected from Sn_1_/V_o_-CuO. (C) LMM Auger spectra of Cu for Sn_1_/V_o_-CuO. (D) High-resolution XPS spectrum of Sn 3d for Sn_1_/V_o_-CuO; Figure S5: Effect of benzaldehyde/1-octene ratio on the epoxide yield among 1-octene epoxidation. Reaction condition: 10 mg catalyst, 1 mmol 1-octene, 5 mL acetonitrile, 60 °C, 12 h, O_2_ balloon; Figure S6: Effect of temperature on the epoxide yield among 1-octene epoxidation. Reaction condition: 10 mg catalyst, 1 mmol 1-octene, 5 mmol benzaldehyde, 5 mL acetonitrile, 12 h, O_2_ balloon; Figure S7: Solvent effect for the epoxidation of 1-octene. Reaction condition: 10 mg catalyst, 1 mmol 1-octene, 5 mmol benzaldehyde, 60 °C, 12 h, O_2_ balloon; Figure S8: XRD spectra of spent Sn_1_/V_o_-CuO and fresh Sn_1_/V_o_-CuO; Figure S9: SEM images of spent Sn_1_/V_o_-CuO; Figure S10: EPR spectra of Mukaiyama epoxidation(anaerobic) and anaerobic oxidation of benzaldehyde; Figure S11:Crystal structure of CuO (O: red, Cu: orange); Figure S12: Side views of (a) CuO(−111), (b) V_o_-CuO(−111) and (c) Sn_1_/V_o_-CuO(−111) surface structures with the oxygen vacancy formation energy Ef(V_o_) (O: red, Cu: orange, Sn: grey); Figure S13: Geometric structures involved in the formation process of benzoyl peroxy radical on CuO(−111). In each elementary step, the initial state, transition state, and final state are presented (O: red, Cu: orange, H: blue, C: black, O in adsorbate: pink); Figure S14: Geometric structures involved in the formation process of benzoyl peroxy radical on Sn_1_/V_o_-CuO(−111). In each elementary step, the initial state, transition state, and final state are presented (O: red, Cu: orange, Sn: grey, H: blue, C: black, O in adsorbate: pink); Figure S15: Geometric structures involved in the formation process of benzoyl peroxy radical on Sn_1_/V_o_-CuO(-111). In each elementary step, the initial state, transition state, and final state are presented (O: red, Cu: orange, Sn: grey, H: blue, C: black, O in adsorbate: pink). Table S1: Catalytic performance of different catalysts; Table S2: Effect of reaction time for epoxidation of 1-octene with Sn_1_/V_o_-CuO; Table S3: Recycling of Sn_1_/V_o_-CuO for 1-octene epoxidation; Table S4: The results for the epoxidation of various olefins; Table S5: The atomic ratio of Cu:Sn in the as-prepared samples measured by ICP-OES tests; Table S6: Structural parameters of Sn_1_/V_o_-CuO-90 extracted from the EXAFS fitting. References [34,42,43,44,45,46,47,48,49,50,51,52] are cited in the Supplementary Materials.

## References

[1] Zhou Z., Wang F., Yan T., Wan H., Yao R., Zhang K., Liu Y., Wang S., Xu D., Hou H. (2022). Boosting the epoxidation of long-chain linear α-olefins via bimetallic CoIr composite. Fuel.

[2] Zăvoianu R., Cruceanu A., Pavel O.D., Bradu C., Florea M., Bîrjega R. (2022). Green Epoxidation of Olefins with Zn_x_Al/Mg_x_Al-LDH Compounds: Influence of the Chemical Composition. Catalysts.

[3] Zhou Z., Zhang K., He P., Chang H., Zhang M., Yan T., Zhang X., Li Y., Cao Z. (2024). Reactive Intermediate Confinement in Beta Zeolites for the Efficient Aerobic Epoxidation of α-Olefins. Angew. Chem. Int. Ed..

[4] Yang X., Li X., Dong J. (2021). Magnesium Pyrophosphate-Catalyzed Epoxidation of 1-Octene with Aqueous Hydrogen Peroxide. Catal. Lett..

[5] Zhang M., Xiang H., Wen X. (2024). Enhancing Catalytic Efficiency in Long-Chain Linear α-Olefin Epoxidation: A Study of CaSnO_3_-Based Catalysts. Catalysts.

[6] Li P., Gao J., Shi J., Wang H., Xing X., Ren J., Meng Y., Wang L., Lv B. (2022). Insights into the effect of oxygen vacancies on the epoxidation of 1-hexene with hydrogen peroxide over WO_3−x_/SBA-15. Catal. Sci. Technol..

[7] Yoshimura Y., Ogasawara Y., Suzuki K., Yamaguchi K., Mizuno N. (2017). “Release and catch” catalysis by tungstate species for the oxidative cleavage of olefins. Catal. Sci. Technol..

[8] Ren J., Wang L., Li P., Xing X., Wang H., Lv B. (2022). Ag supported on alumina for the epoxidation of 1-hexene with molecular oxygen: The effect of Ag^+^/Ag^0^. New J. Chem..

[9] Triandafillidi I., Kokotou M.G., Lotter D., Sparr C., Kokotos C.G. (2021). Aldehyde-catalyzed epoxidation of unactivated alkenes with aqueous hydrogen peroxide. Chem. Sci..

[10] Yang X., Li X., Dong J. (2021). Farringtonite as an efficient catalyst for linear-chain α-olefin epoxidation with aqueous hydrogen peroxide. New J. Chem..

[11] Florio S., Troisi L., Capriati V., Coletta G. (1999). Heterosubstituted chlorohydrins: Knoevenagel reactionversus epoxide formation. Tetrahedron Lett..

[12] Domask W., Kobe K. (1954). Synthesis of Ethylene Chlorohydrin. Ind. Eng. Chem..

[13] Yang H., Liu Q., Li Y., Sun K., Chen Z., Peng Q., Chen C. (2020). Isolated Single-Atom Ruthenium Anchored on Beta Zeolite as an Efficient Heterogeneous Catalyst for Styrene Epoxidation. ChemNanoMat.

[14] Mello R., Alcalde-Aragonés A., González Núñez M.E., Asensio G. (2012). Epoxidation of Olefins with a Silica-Supported Peracid. J. Org. Chem..

[15] Zhou X., Ji H. (2010). Biomimetic kinetics and mechanism of cyclohexene epoxidation catalyzed by metalloporphyrins. Chem. Eng. J..

[16] Gong M., Guo Y., Malko D., Rubio-Garcia J., Dawson J.M.S., Britovsek G.J.P., Kucernak A. (2022). Using molecular oxygen and Fe–N/C heterogeneous catalysts to achieve Mukaiyama epoxidations via in situ produced organic peroxy acids and acylperoxy radicals. Catal. Sci. Technol..

[17] Yuan Q., Hagen A., Roessner F. (2006). An investigation into the Ti-grafting structure on MCM-41 and epoxidation catalysis. Appl. Catal. A Gen..

[18] Rezaeifard A., Haddad R., Jafarpour M., Hakimi M. (2013). Catalytic Epoxidation Activity of Keplerate Polyoxomolybdate Nanoball toward Aqueous Suspension of Olefins under Mild Aerobic Conditions. J. Am. Chem. Soc..

[19] Qian L., Wang Z., Beletskiy E.V., Liu J., dos Santos H.J., Li T., Rangel M.d.C., Kung M.C., Kung H.H. (2017). Stable and solubilized active Au atom clusters for selective epoxidation of cis-cyclooctene with molecular oxygen. Nat. Commun..

[20] Pattisson S., Nowicka E., Gupta U.N., Shaw G., Jenkins R.L., Morgan D.J., Knight D.W., Hutchings G.J. (2016). Tuning graphitic oxide for initiator- and metal-free aerobic epoxidation of linear alkenes. Nat. Commun..

[21] Gupta U.N., Dummer N.F., Pattisson S., Jenkins R.L., Knight D.W., Bethell D., Hutchings G.J. (2014). Solvent-Free Aerobic Epoxidation of Dec-1-ene Using Gold/Graphite as a Catalyst. Catal. Lett..

[22] Engel R.V., Alsaiari R., Nowicka E., Miedziak P.J., Kondrat S.A., Morgan D.J., Edwards J.K., Hutchings G.J. (2019). Solvent-free aerobic epoxidation of 1-decene using supported cobalt catalysts. Catal. Today.

[23] Aprile C., Corma A., Domine M.E., Garcia H., Mitchell C. (2009). A cascade aerobic epoxidation of alkenes over Au/CeO_2_ and Ti-mesoporous material by “in situ” formed peroxides. J. Catal..

[24] Xu D., He Y., Liu X., Xiong C., Zhou X., Xue C., Ji H. (2021). N-Hydroxyphthalimide-Catalyzed Epoxidation of Inactive Aliphatic Olefins with Air at Room Temperature. Asian J. Org. Chem..

[25] Muratsugu S., Weng Z., Tada M. (2013). Surface Functionalization of Supported Mn Clusters to Produce Robust Mn Catalysts for Selective Epoxidation. ACS Catal..

[26] Tada M., Muratsugu S., Kinoshita M., Sasaki T., Iwasawa Y. (2010). Alternative Selective Oxidation Pathways for Aldehyde Oxidation and Alkene Epoxidation on a SiO_2_-Supported Ru-Monomer Complex Catalyst. J. Am. Chem. Soc..

[27] Sepelak V., Begin-Colin S., Le Caer G. (2012). Transformations in oxides induced by high-energy ball-milling. Dalton Trans.

[28] Madadi S., Bergeron J.-Y., Kaliaguine S. (2021). Kinetic investigation of aerobic epoxidation of limonene over cobalt substituted mesoporous SBA-16. Catal. Sci. Technol..

[29] Hu R., Yang P., Pan Y., Li Y., He Y., Feng J., Li D. (2017). Synthesis of a highly dispersed CuO catalyst on CoAl-HT for the epoxidation of styrene. Dalton Trans.

[30] Scotti N., Ravasio N., Zaccheria F., Psaro R., Evangelisti C. (2013). Epoxidation of alkenes through oxygen activation over a bifunctional CuO/Al_2_O_3_ catalyst. Chem. Commun..

[31] Zhang W., Zhu N., Ding L., Hu Y., Wu Z. (2021). Efficacious CO_2_ Adsorption and Activation on Ag Nanoparticles/CuO Mesoporous Nanosheets Heterostructure for CO_2_ Electroreduction to CO. Inorg. Chem..

[32] Tran T.H., Nguyen M.H., Nguyen T.H.T., Dao V.P.T., Nguyen P.M., Nguyen V.T., Pham N.H., Le V.V., Sai C.D., Nguyen Q.H. (2019). Effect of annealing temperature on morphology and structure of CuO nanowires grown by thermal oxidation method. J. Cryst. Growth.

[33] Costa I.M., Colmenares Y.N., Pizani P.S., Leite E.R., Chiquito A.J. (2018). Sb doping of VLS synthesized SnO_2_ nanowires probed by Raman and XPS spectroscopy. Chem. Phys. Lett..

[34] Zhong X., Liang S., Yang T., Zeng G., Zhong Z., Deng H., Zhang L., Sun X. (2022). Sn Dopants with Synergistic Oxygen Vacancies Boost CO_2_ Electroreduction on CuO Nanosheets to CO at Low Overpotential. ACS Nano.

[35] Chu S., Kang C., Park W., Han Y., Hong S., Hao L., Zhang H., Lo T.W.B., Robertson A.W., Jung Y. (2022). Single atom and defect engineering of CuO for efficient electrochemical reduction of CO_2_ to C_2_H_4_. SmartMat.

[36] Wei Z., Zhao M., Yang Z., Duan X., Jiang G., Li G., Zhang F., Hao Z. (2023). Oxygen vacancy-engineered titanium-based perovskite for boosting H_2_O activation and lower-temperature hydrolysis of organic sulfur. Proc. Natl. Acad. Sci. USA.

[37] Cao J., Zhou Z., Zhang M., Gong N., Yin A., Cai Y., Sun X., Wan H., Li Y., Cao Z. (2023). Tuning the electronic properties of supported Cu catalysts for efficient epoxidation of long-chain α-olefins. Fuel.

[38] Marvilliers A., Harlé J.-B., Launay F., Blanchard S., Gauvin-Bialecki A., Gérard H., Villanneau R. (2024). Investigating the Mukaiyama-type epoxidation reaction of alkenes with Cr(III) salophen catalysts: A confrontation between experiments and DFT calculations. J. Catal..

[39] Liu J., Tang H., Jian P., Liu B. (2023). Oxygen-vacancy defect engineering to boost the aerobic oxidation of limonene over Co_3_O_4_ nanocubes. Appl. Catal. B Environ..

[40] Nam W., Kim H.J., Kim S.H., Ho R.Y.N., Valentine J.S. (1996). Metal Complex-Catalyzed Epoxidation of Olefins by Dioxygen with Co-Oxidation of Aldehydes. A Mechanistic Study. Inorg. Chem..

[41] Wentzel B.B., Alsters P.L., Feiters M.C., Nolte R.J.M. (2004). Mechanistic Studies on the Mukaiyama Epoxidation. J. Org. Chem..

[42] Kresse G., Furthmüller J. (1996). Efficiency of ab-initio total energy calculations for metals and semicoasis set. Comput. Mater. Sci..

[43] Kresse G., Furthmüller J. (1996). Efficient iterative schemes for ab initio total-energy calculations using a plane-wave basis set. Phys. Rev. B.

[44] Blöchl P.E. (1994). Projector augmented-wave method. Phys. Rev. B.

[45] Kresse G., Joubert D. (1999). From ultrasoft pseudopotentials to the projector augmented-wave method. Phys. Rev. B.

[46] Perdew J.P., Burke K., Ernzerhof M. (1996). Generalized Gradient Approximation Made Simple. Phys. Rev. Lett..

[47] Grimme S., Antony J., Ehrlich S., Krieg H. (2010). A consistent and accurate ab initio parametrization of density functional dispersion correction (DFT-D) for the 94 elements H-Pu. J. Chem. Phys..

[48] Grimme S., Ehrlich S., Goerigk L. (2011). Effect of the damping function in dispersion corrected density functional theory. J. Comput. Chem..

[49] Henkelman G., Uberuaga B.P., Jónsson H. (2000). A climbing image nudged elastic band method for finding saddle points and minimum energy paths. J. Chem. Phys..

[50] Zhou C.-Y., Wang D., Gong X.-Q. (2019). A DFT+U revisit of reconstructed CeO_2_(100) surfaces: Structures, thermostabilities and reactivities. Phys. Chem. Chem. Phys..

[51] Wang V., Xu N., Liu J.-C., Tang G., Geng W.-T. (2021). VASPKIT: A user-friendly interface facilitating high-throughput computing and analysis using VASP code. Comput. Phys. Commun..

[52] Guo W., Liu S., Tan X., Wu R., Yan X., Chen C., Zhu Q., Zheng L., Ma J., Zhang J. (2021). Highly Efficient CO_2_ Electroreduction to Methanol through Atomically Dispersed Sn Coupled with Defective CuO Catalysts. Angew. Chem. Int. Ed..

