# Supplementary material for: Enhanced Catalytic Performance of Sn Single-Atom Doped CuO with Oxygen Vacancies for Efficient Epoxidation of α-Olefins
Source: Molecules. 2025 Feb 25;30(5):1042. doi: 10.3390/molecules30051042 (PMC11901971; doi:10.3390/molecules30051042)
Supplement: Supplementary file 1 [file molecules-30-01042-s001.zip › molecules-3431319-supplementary.pdf]

Supplementary Information

Enhanced Catalytic Performance of Sn Single-Atom Doped CuO with Oxygen Vacancies for Efficient Epoxidation of α-Olefins

Min Zhang [1,2,†], Gaolei Qin [1,2,†], Pengfei Li [2,], Xiangjie Zhang [1,2], Hongying Chang [2], Ziyu Zhou [1,2], Wei Zhao [1,2], Xumeng Huang [1,2], Kui Tang [1,2], Yonghe Ning [1], Chang Song [1,*] and Peng He [1,2,*]

[1] State Key Laboratory of Coal Conversion, Institute of Coal Chemistry, Chinese Academy of Sciences, Taiyuan 030001, China; zhangmin@sxicc.ac.cn (M.Z.); qingaolei21@mails.ucas.ac.cn (G.Q.); zhangxiangjie18@mails.ucas.ac.cn (X.Z.); zhouziyu@sxicc.ac.cn (Z.Z.); zhaowei22@mails.ucas.ac.cn (W.Z.); huangxumeng24@mails.ucas.ac.cn (X.H.); tangkui24@mails.ucas.ac.cn (K.T.); ningyongh@163.com (Y.N.)

[2] School of Chemical Engineering, University of Chinese Academy of Sciences, Beijing 100049, China; lipengfei21@mails.ucas.ac.cn (P.L.); changhongying22@mails.ucas.ac.cn (H.C.)

* Correspondence: songchang@sxicc.ac.cn (C.S.); hepeng@sxicc.ac.cn (P.H.)

† These authors contributed equally to this work.

Methods

All calculations were performed using the plane-wave-based periodic DFT method implemented in the Vienna ab initio simulation package (VASP),[1,2] where the ionic cores were described by the projector augmented wave (PAW) method.[3,4] The exchange and correlation energies were computed using the Perdew-Burke-Ernzerhof functional(PBE).[5] The van der Waals interactions were considered using the DFT-D3 semiempirical method.[6,7] To achieve accurate energies with errors of less than 1 meV per atom, the cutoff energy was set at 400 eV. The Gaussian electron smearing method with σ = 0.05 eV and ISMEAR = 0 were used. Geometric optimization converged until the forces acting on the atoms were smaller than 0.03 eV·Å^{-1}, whereas the energy threshold-defining self-consistency of the electron density was set to 10^{-5} eV. Spin polarization was included to correctly describe magnetic properties, which is essential for an accurate description of all energetic data. All transition state structures were optimized by using the climbing image nudged elastic band (CI-NEB) method,[8] and the frequency analysis was also processed to verify an authentic transition state having only one imaginary frequency and provides zero-point energy (ZPE). The reaction barrier (E_a) and reaction energy (E_r) are calculated according to $E_a = E_{TS} - E_{IS}$ and $E_r = E_{FS} - E_{IS}$, where E_{IS}, E_{FS} and E_{TS} are the energies of the corresponding initial state (IS), final state (FS), and transition state (TS), respectively. Therefore, the more negative the E_a and E_r, the easier for this reaction. The oxygen vacancy formation energy $E_f(V_O)$ was defined as $E_f(V_O) = E(V_O\text{-slab}) + 1/2E(O_2) - E(\text{slab})$, where $E(V_O\text{-slab})$ and $E(O_2)$ are the total energies of the slab with a single V_O and an O_2 molecule in the gas phase, respectively.[9] For the calculations on the Gibbs free energies, the zero-point energy (ZPE) and entropy contributions were included according to the following equation, $\Delta G = \Delta E + \Delta ZPE - T\Delta S$, which were calculated based on the molecular vibration analysis (333 K) by vaspkit.1.3.3.[10]

Models

The models utilized in our theoretical calculations were based on the experimental characterization results (XRD, Figure 1B). The oxygen-terminated CuO(−111) surface was cleaved from the CuO (a = 4.65 Å, b = 3.41 Å, c = 5.11 Å, Figure S12) crystal structure from the database of the material project. An O-Cu-O three layers periodic (2 × 4) supercell surface model was constructed with all atoms fully relaxed during optimization (Figure S13a). The oxygen vacancy modified surface V_O-CuO(−111) (Figure S13b) was built by removing one terminal O atom on the perfect CuO(−111) surface. Sn-doped Sn/V_O-CuO(−111) with O vacancy (Figure S13c) was built by replacing one Cu with Sn and removing one O on the CuO(−111) surface. A (2 × 2 × 1) k-point grid was utilized for sampling the Brillouin zone, and a 15 Å vacuum was introduced between the repeated slabs along the z-direction to avoid significant interactions. The similar surfaces had also been reported in previous work with the same models and methods as we utilized in our work.[11,12]

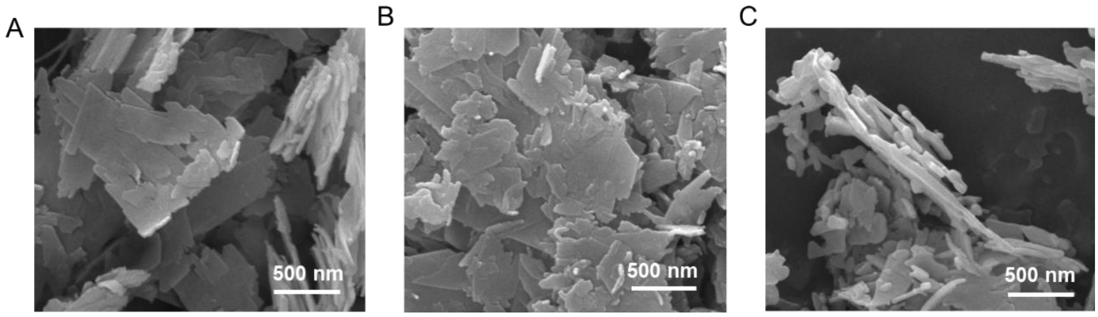

Figure S1. SEM images of (A) CuO, (B) V_o-CuO, (C) Sn/CuO nanosheets.

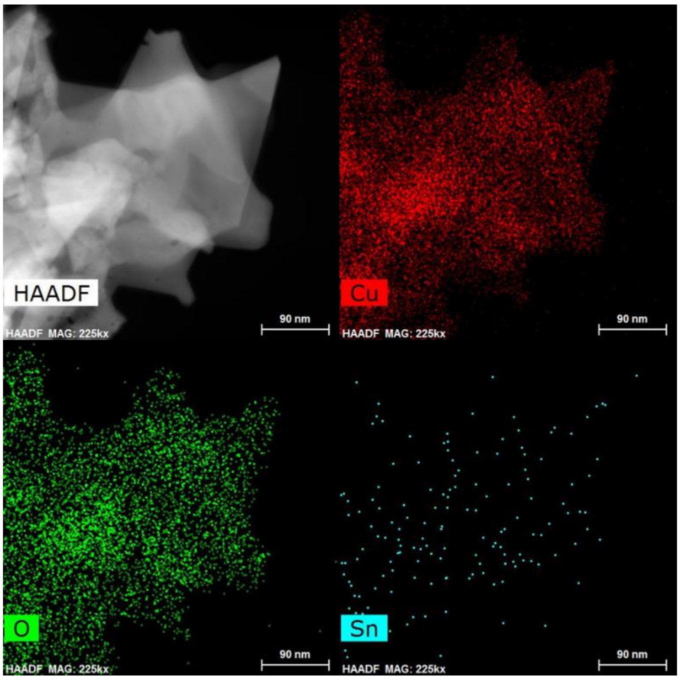

Figure S2. EDS spectrum of the as-synthesized Sn/ CuO.

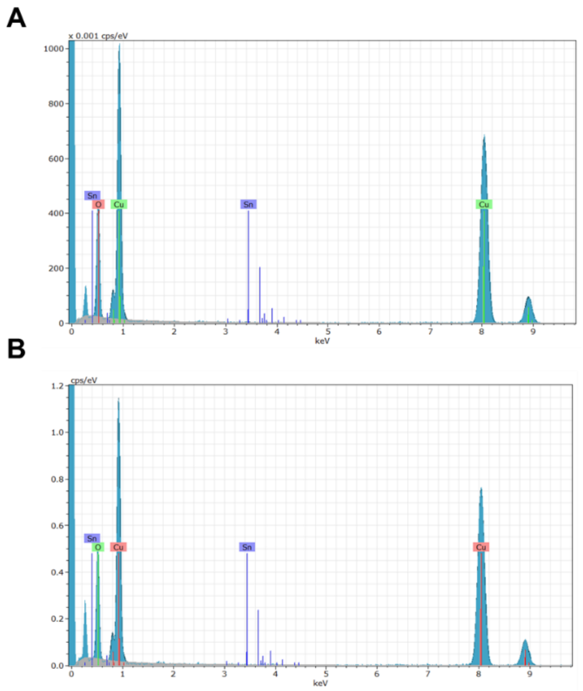

Figure S3. (A) EDS spectrum of the as-synthesized Sn/CuO. (B) EDS spectrum of the as-synthesized Sn_1/V_o-CuO.

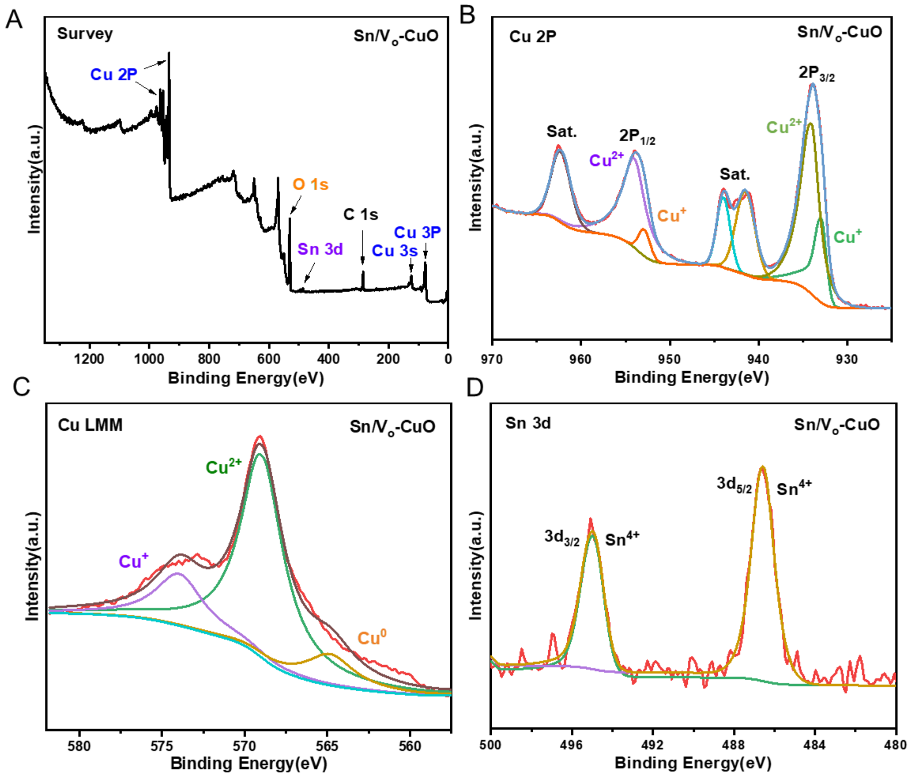

Figure S4. (A) XPS survey spectrum of Sn_1/V_o-CuO nanosheets. (B) High-resolution XPS spectrum of Cu 2p collected from Sn_1/V_o-CuO.(C) LMM Auger spectra of Cu for Sn_1/V_o-CuO.(D) High-resolution XPS spectrum of Sn 3d for Sn_1/V_o-CuO.

Table S1 Catalytic performance of different catalysts.

Catalysts	Conversion (%)	Selectivity (%)	Yield (%)
SnO_2	3.8	18.4	0.7
CuO	40.5	95.1	38.5
V_o-CuO	77.3	93.5	72.3
Sn/CuO	55.46	94.8	52.6
Sn/V_o-CuO	97.2	95.6	92.9

Table S2 Effect of reaction time for epoxidation of 1-octene with Sn_1/V_o-CuO.

Time (h)	Conversion (%)	Selectivity (%)	Yield (%)
3	42	96.0	40.3
6	70	95.3	66.7
9	85	95.1	80.8
12	97.2	95.6	92.9
15	99.2	95.9	95.1

Table S3 Recycling of Sn1/Vo-CuO for 1-octene epoxidation.

Cycles	Conversion (%)	Selectivity (%)	Yield (%)
1	97.2	95.6	92.9
2	98.2	93.9	92.2
3	97.5	94.9	92.5
4	97.1	94.7	92.0
5	97.7	94.7	92.5
6	97.3	95.0	92.4

Table S4 The results for the epoxidation of various olefins.

Substrates	Conversion (%)	Selectivity (%)	Yield (%)
1-hexene	97.9	97.0	95.0
1-decene	99.2	95.6	94.8
1-dodecene	98.4	95.6	94.1
1-teradecene	99.1	95.4	94.5

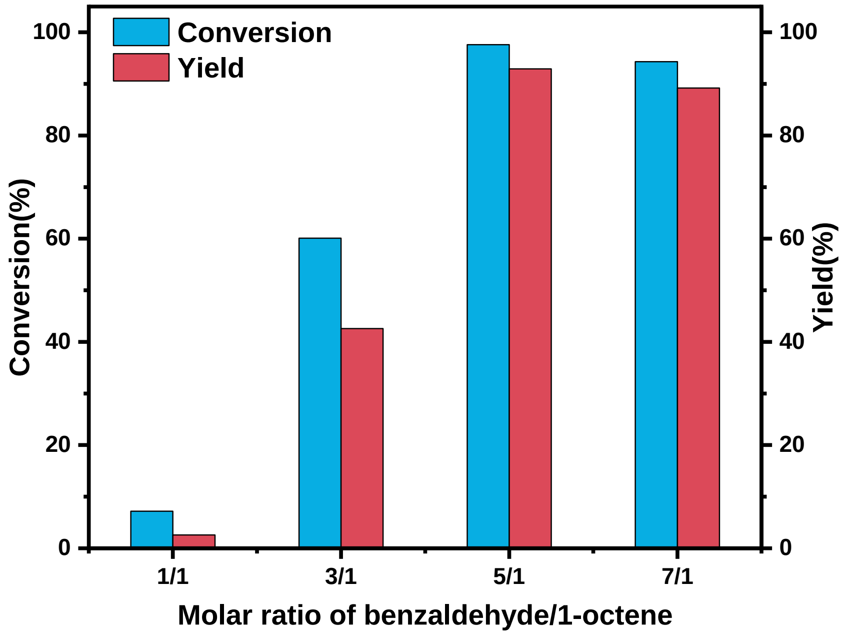

Figure S5. Effect of benzaldehyde /1-octene ratio on the epoxide yield among 1-octene epoxidation. Reaction condition: 10mg catalyst, 1 mmol 1-octene, 5 mL acetonitrile, 60 °C, 12 h, O_2 balloon.

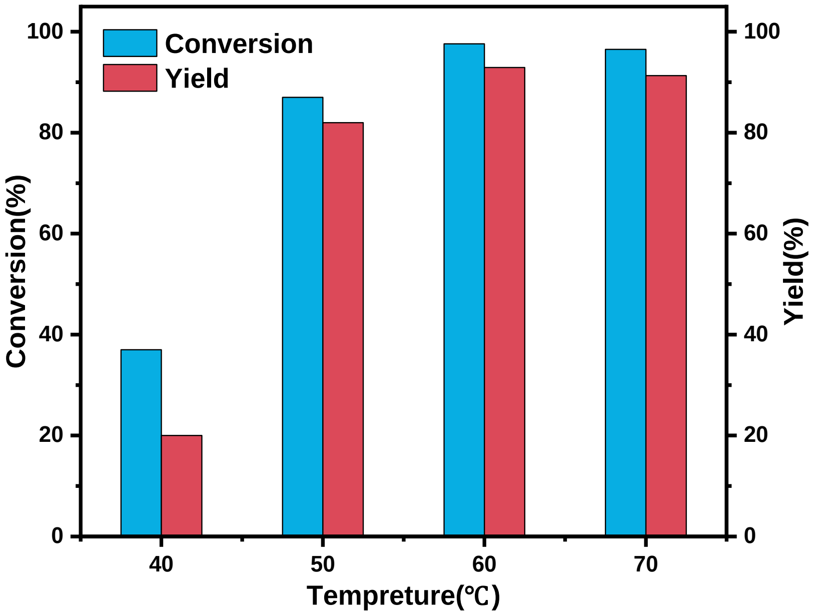

Figure S6. Effect of temperature on the epoxide yield among 1-octene epoxidation. Reaction condition: 10mg catalyst, 1 mmol 1-octene, 5 mmol benzaldehyde, 5 mL acetonitrile, 12 h, O_2 balloon.

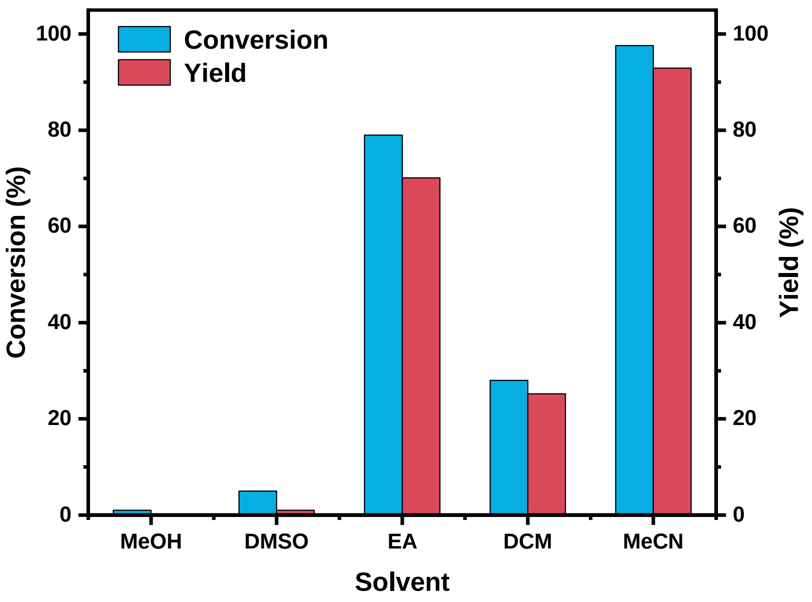

Figure S7. Solvent effect for the epoxidation of 1-octene. Reaction condition: 10mg catalyst, 1 mmol 1-octene, 5 mmol benzaldehyde, 60 °C, 12 h, O_2 balloon.

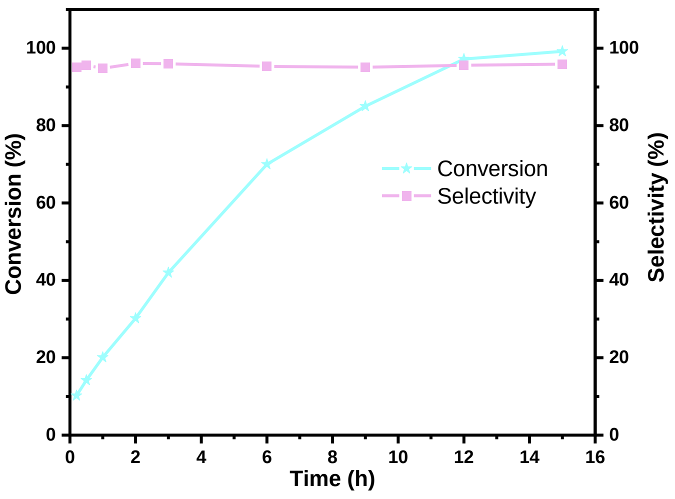

Figure S8. Time course of substrate conversion and products selectivity for epoxidation of 1-octene.

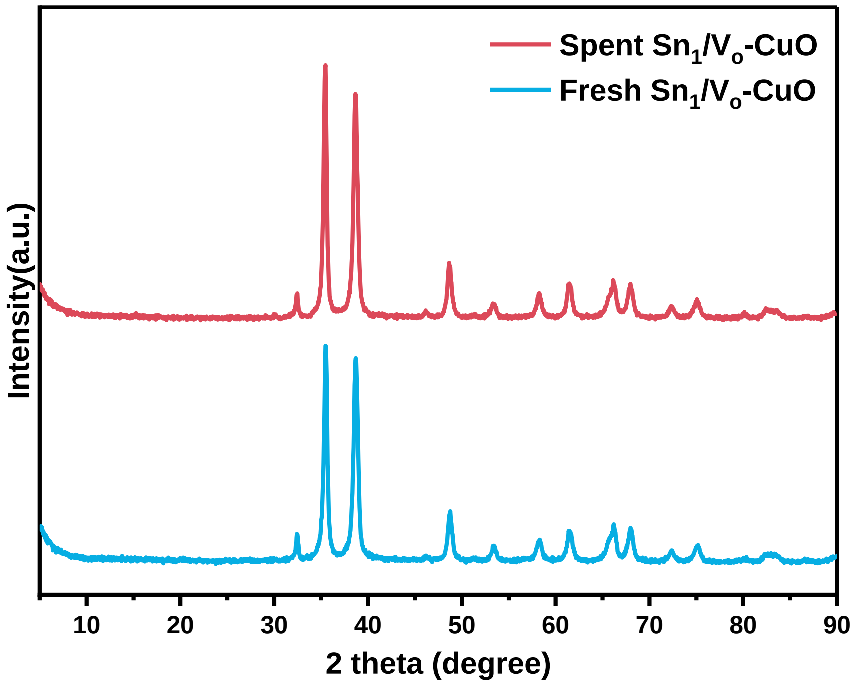

Figure S9. XRD spectra of spent Sn_1/V_o-CuO and fresh Sn_1/V_o-CuO.

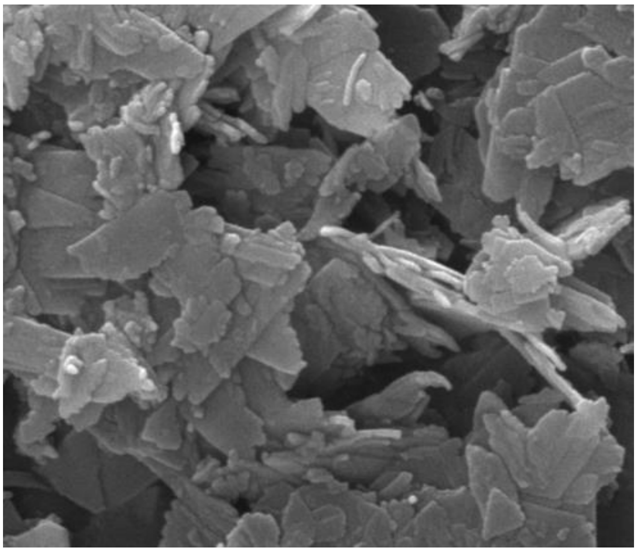

Figure S10. SEM image of spent Sn_1/V_o-CuO.

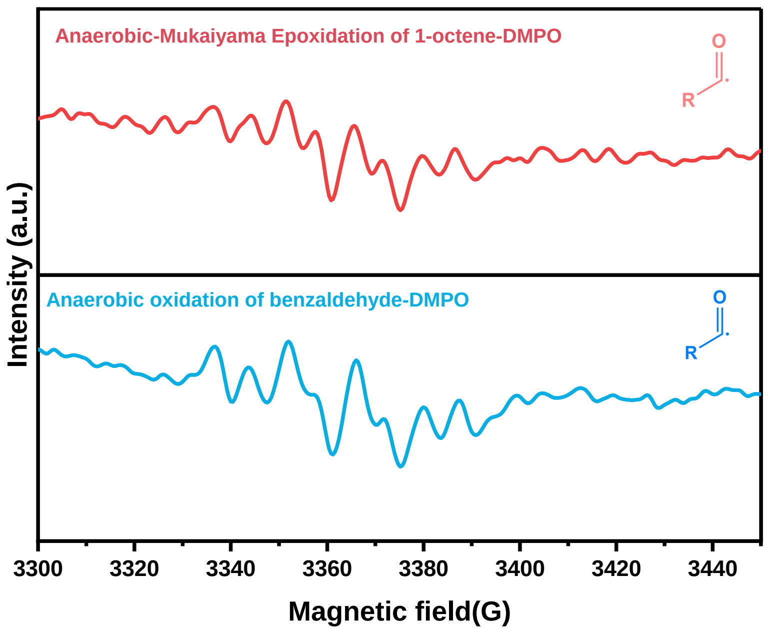

Figure S11. EPR spectra of Mukaiyama epoxidation(anaerobic) and anaerobic oxidation of benzaldehyde.

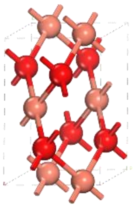

Figure S12. Crystal structure of CuO (O: red, Cu: orange).

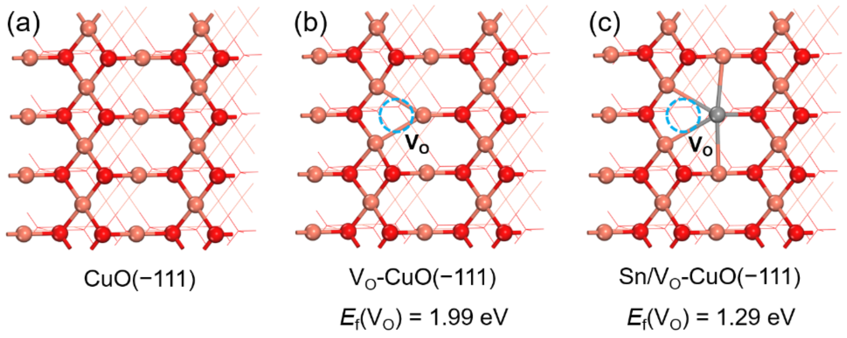

Figure S13. Side views of (a) CuO(−111), (b) V_O-CuO(−111) and (c) Sn/V_O-CuO(−111) surface structures with the oxygen vacancy formation energy $E_f(V_O)$ (O: red, Cu: orange, Sn: grey).

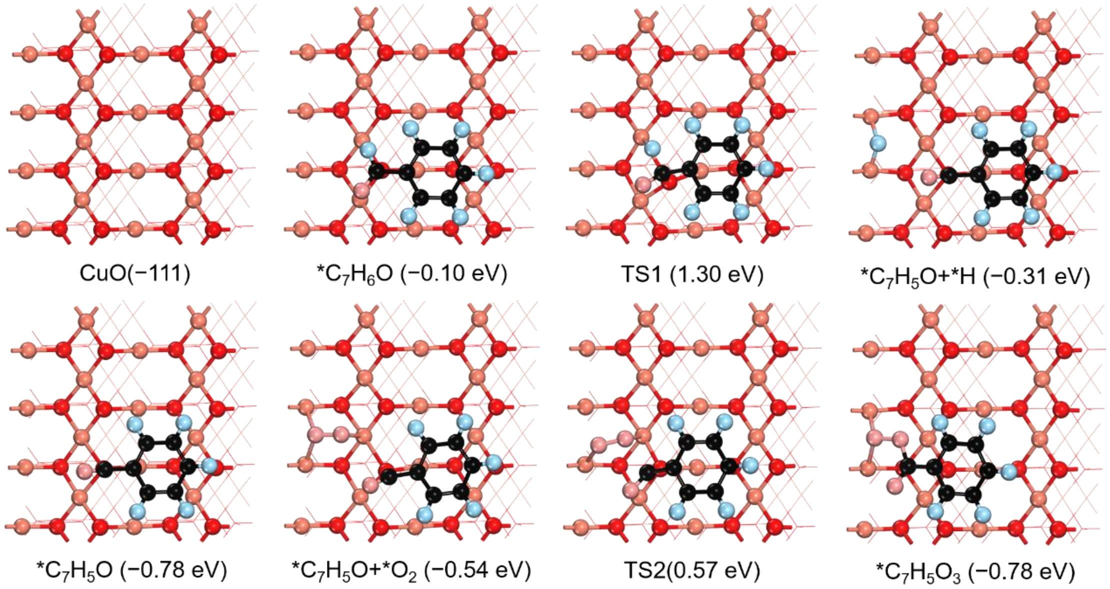

Figure S14. Geometric structures involved in the formation process of benzoyl peroxy radical on CuO(−111). In each elementary step, the initial state, transition state and final state are presented (O: red, Cu: orange, H: blue, C: black, O in adsorbate: pink).

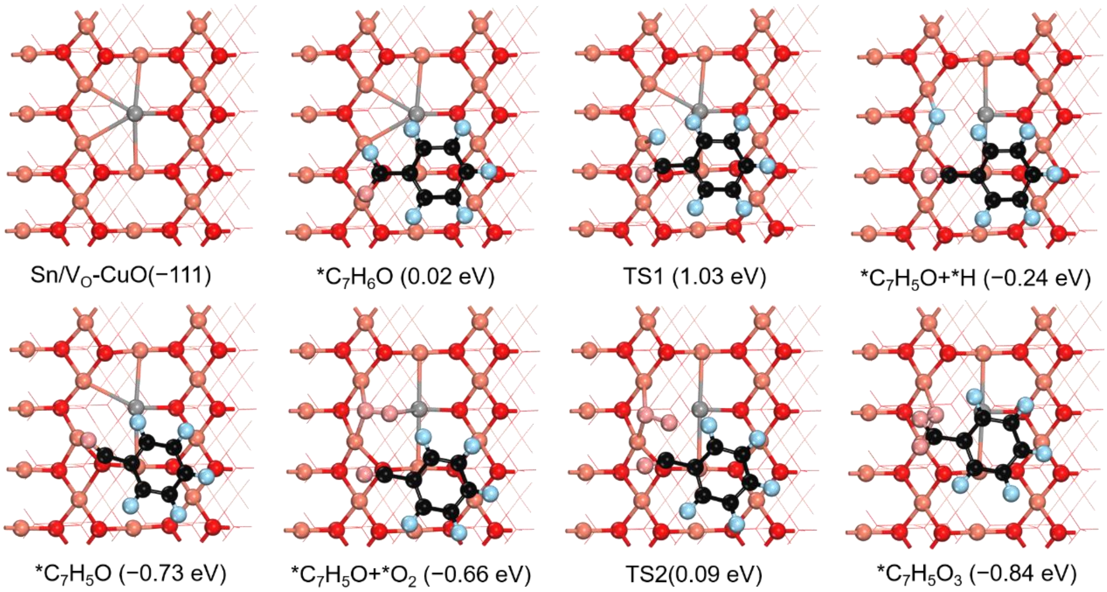

Figure S15. Geometric structures involved in the formation process of benzoyl peroxy radical on Sn_1/V_o-CuO(−111). In each elementary step, the initial state, transition state and final state are presented (O: red, Cu: orange, Sn: grey, H: blue, C: black, O in adsorbate: pink).

Table S5. The atomic ratio of Cu : Sn in the as-prepared samples measured by ICP-OES tests.

Sample	[Cu] (μg/g)	[Sn] (μg /g)	Atomic ratio (%) Cu:Sn
Sn/CuO	869000	1587	99.82 : 0.18
Sn_1/V_o-CuO	841000	1745	99.79 : 0.21

Table S6. Structural parameters of the catalysts extracted from the EXAFS fitting.

Sample	path	CN	R(Å)	$\sigma^2/10^{-3}$(Å^2)
V_o-CuO	Cu-O	3.86	1.94	3.86
Sn_1/V_o-CuO	Cu-O	3.71	1.95	3.71

Reference

1. Kresse, G.; Furthmüller, J. Efficiency of ab-initio total energy calculations for metals and semiconductors using a plane-wave basis set. Computational Materials Science 1996, 6, 15-50, doi:https://doi.org/10.1016/0927-0256(96)00008-0.
2. Kresse, G.; Furthmüller, J. Efficient iterative schemes for ab initio total-energy calculations using a plane-wave basis set. Physical Review B 1996, 54, 11169-11186, doi:10.1103/PhysRevB.54.11169.
3. Blöchl, P.E. Projector augmented-wave method. Physical Review B 1994, 50, 17953-17979, doi:10.1103/PhysRevB.50.17953.
4. Kresse, G.; Joubert, D. From ultrasoft pseudopotentials to the projector augmented-wave method. Physical Review B 1999, 59, 1758-1775, doi:10.1103/PhysRevB.59.1758.
5. Perdew, J.P.; Burke, K.; Ernzerhof, M. Generalized Gradient Approximation Made Simple. Physical Review Letters 1996, 77, 3865-3868, doi:10.1103/PhysRevLett.77.3865.
6. Grimme, S.; Antony, J.; Ehrlich, S.; Krieg, H. A consistent and accurate ab initio parametrization of density functional dispersion correction (DFT-D) for the 94 elements H-Pu. J Chem Phys 2010, 132, 154104, doi:10.1063/1.3382344.
7. Grimme, S.; Ehrlich, S.; Goerigk, L. Effect of the damping function in dispersion corrected density functional theory. J Comput Chem 2011, 32, 1456-1465, doi:10.1002/jcc.21759.
8. Henkelman, G.; Uberuaga, B.P.; Jónsson, H. A climbing image nudged elastic band method for finding saddle points and minimum energy paths. The Journal of Chemical Physics 2000, 113, 9901-9904, doi:10.1063/1.1329672.
9. Zhou, C.-Y.; Wang, D.; Gong, X.-Q. A DFT+U revisit of reconstructed CeO_2(100) surfaces: structures, thermostabilities and reactivities. Physical Chemistry Chemical Physics 2019, 21, 19987-19994, doi:10.1039/c9cp03408k.
10. Wang, V.; Xu, N.; Liu, J.-C.; Tang, G.; Geng, W.-T. VASPKIT: A user-friendly interface facilitating high-throughput computing and analysis using VASP code. Computer Physics Communications 2021, 267, 108033, doi:https://doi.org/10.1016/j.cpc.2021.108033.
11. Zhong, X.; Liang, S.; Yang, T.; Zeng, G.; Zhong, Z.; Deng, H.; Zhang, L.; Sun, X. Sn Dopants with Synergistic Oxygen Vacancies Boost CO_2 Electroreduction on CuO Nanosheets to CO at Low Overpotential. ACS Nano 2022, 16, 19210-19219, doi:10.1021/acsnano.2c08436.
12. Guo, W.; Liu, S.; Tan, X.; Wu, R.; Yan, X.; Chen, C.; Zhu, Q.; Zheng, L.; Ma, J.; Zhang, J.; et al. Highly Efficient CO_2 Electroreduction to Methanol through Atomically Dispersed Sn Coupled with Defective CuO Catalysts. Angewandte Chemie International Edition 2021, 60, 21979-21987, doi:10.1002/anie.202108635.
